# Evaluation of a community-based hypertension improvement program (ComHIP) in Ghana: data from a baseline survey

**DOI:** 10.1186/s12889-017-4260-5

**Published:** 2017-04-28

**Authors:** Peter Lamptey, Amos Laar, Alma J. Adler, Rebecca Dirks, Aya Caldwell, David Prieto-Merino, Ann Aerts, Neil Pearce, Pablo Perel

**Affiliations:** 1 Department of Non-communicable Disease Epidemiology, London School of Hygiene & Tropical Medicine, Keppel St, London, WC1E 7HT UK; 2 Department of Population, Family, & Reproductive Health, School of Public Health, University of Ghana, LG 13, Legon, Accra, Ghana; 3 Family Health International 360, Washington DC, WA USA; 4 Novartis Foundation, Basel, Switzerland; 5 Applied Statistical Methods Research Group, Universidad Catolica San Antonio de Murcia (UCAM), Murcia, Spain

## Abstract

**Background:**

Ghana faces an increasing burden of non-communicable disease with rates of hypertension estimated as high as 36% in adults. Despite these high rates, hypertension control remains very poor in Ghana (4%). The current project aims to implement and evaluate a community-based programme to raise awareness, and to improve treatment and control of hypertension in the Eastern Region of Ghana. In this paper, we present the findings of the baseline cross-sectional survey focusing on hypertension prevalence, awareness, treatment, and control.

**Methods:**

To evaluate the ComHIP project, a quasi-experimental design consisted of a before and after evaluations are being implemented in the intervention and comparison districts. A cohort study component is being implemented in the intervention district to assess hypertension control. Background anthropometric and clinical data collected as part of the baseline survey were analyzed in STATA Version 11. We examined the characteristics of individuals, associated with the baseline study outcomes using logistic regression models.

**Results:**

We interviewed 2400 respondents (1200 each from the comparison and intervention districts), although final sample sizes after data cleaning were 1170 participants in the comparison district and 1167 in the intervention district. With the exception of ethnicity, the control and intervention districts compare favorably. Overall 32.4% of the study respondents were hypertensive (31.4% in the control site; and 33.4% in the intervention site); 46.2% of hypertensive individuals were aware of a previous diagnosis of hypertension (44.7% in the control site, and 47.7% in the intervention site), and only around 9% of these were being treated in either arm. Hypertension control was 1.3% overall (0.5% in the comparison site, and 2.1% in the intervention site). Age was a predictor of having hypertension, and so was increasing body mass index (BMI), waist, and hip circumferences. After adjusting for age, the risk factors with the greatest association with hypertension were being overweight (aOR = 2.30; 95% CI 1.53–3.46) or obese (aOR = 3.61; 95% CI 2.37–5.51). Older individuals were more likely to be aware of their hypertension status than younger people. After adjusting for age people with a family history of hypertension or CVD, or having an unhealthy waist hip ratio, were more likely to be aware of their hypertension status.

**Conclusions:**

The high burden of hypertension among the studied population, coupled with high awareness, yet very low level of hypertension treatment and control requires in-depth investigation of the bottlenecks to treatment and control. The low hypertension treatment and control rates despite current and previous general educational programs particularly in the intervention district, may suggest that such programs are not necessarily impactful on the health of the population.

**Electronic supplementary material:**

The online version of this article (doi:10.1186/s12889-017-4260-5) contains supplementary material, which is available to authorized users.

## Background

Available evidence shows raised blood pressure to be one of the leading causes of cardiovascular disease (CVD) and deaths globally, with latest estimates at nearly 10.4 million deaths per year worldwide [1]. The level of raised blood pressure for which treatments have been shown to reduce clinical events in randomized trials is generally accepted as ≥140 systolic mmHg or ≥90 diastolic mm Hg. This level is termed ‘hypertension’ [2]. For individuals diagnosed with hypertension, lowering blood pressure with drugs reduces the risk of subsequent cardiovascular events, including a 35–40% reduction in the risk of stroke and a 20–25% reduction in the risk of myocardial infarction and heart failure [3–5].

Studies show that the current prevalence of hypertension in many low- and middle-income countries (LMICs), particularly in urban societies, is already as high as or higher than in high-income countries [6–8]. Worldwide the prevalence of hypertension in adults ranges between 32 and 50%, with evidence suggesting that the prevalence is increasing in Sub- Saharan Africa [8].

Like many LMICs, Ghana is facing an increasing burden of non-communicable diseases (NCDs), with hypertension at the forefront. The Ghana healthcare system has traditionally focused on addressing communicable diseases and maternal and child health challenges. There is now a great need to build the capacity of existing public and private healthcare systems to improve hypertension screening and management. A decade old report of the Ghana Ministry of Health (MOH) report showed hypertension as the second leading cause of outpatient morbidity in adults older than 45 years [9]. Recent estimates of prevalence of hypertension in Ghana range from 24 to 28% among women and 20–32% among men [10–13]. Of note, the overall rates of hypertension from other local studies [10, 13] are relatively low, in part due to inclusion of younger participants in samples. Hypertension, nonetheless, is a serious health problem in Ghana; common risk factors include increasing body mass index (BMI), increased salt consumption, family history of hypertension and excessive alcohol intake [10, 14].

Studies suggest that the majority of individuals with hypertension are unaware of their status, even fewer are treated for the condition and only a proportion of those have their blood pressure (BP) under control (i.e. blood pressure < 140/90 mmHg) [15, 16]. Awareness of hypertension in Ghana is estimated to range between 16.4 and 54.1% [10, 12, 13, 17] and only 1.7–12.7% in individuals who have their hypertension under control [10, 12, 17]. In the most recent survey involving a nationally representative sample, the level of awareness and treatment status of women and men classified as hypertensive, was alarmingly low. More than 6 in 10 women (63%) and 8 in 10 men (86%) having high blood pressure, reported to be unaware of their condition. Amongst the hypertensive patients, only 17% of the women and 6% of the men were treated and controlled [13].

### The context of hypertension control in Ghana

Ghana’s national NCD Control Programme (NCDCP), under the Disease Control Prevention Department, was established in 1992. The Ghana Health Service (GHS) has developed various guidelines for NCDs and prioritized hypertension. The guidelines recommend that all patients with hypertension be referred for further assessment.

The capacity of the GHS workforce to diagnose and manage hypertension is low. For instance, while training on NCD management is incorporated in the basic nurse education curricula, there is no follow-up in-service training or specialist training on NCD management for nurses. Nurses are currently also not expected to manage hypertension and have to learn on-the-job while assisting specialists.

In partnership with the GHS and the Ghana Police Service, FHI 360 developed a facility- and community-based prevention and screening pilot program for cardiovascular diseases (CVD) in 2009. The program included two sites – the Police Hospital, an urban tertiary hospital located in Ghana’s capital city, Accra, and its surrounding community, and Atua Hospital, a district hospital serving a semi-urban community. A population-based Assessment of Biological and Behavioral NCD risk factors within the pilot communities and a Baseline Health Facility Assessment were completed. CVD screening started in mid-August 2011 and included 14,000 patients by May 2012. Counseling and education on healthy eating and lifestyle were provided alongside screening, which was supported through mHealth lifestyle messages, appointment reminders, and treatment adherence support. Baseline findings of this pilot indicate that hypertension was an important health problem in the areas with prevalence of 31.9% among females and 34.1% among males [18].

**Fig. 1:**
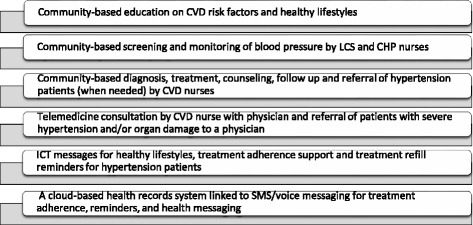
Components of ComHIP

The current paper presents baseline data from a 2-year hypertension control project, the Community-based Hypertension Improvement Project (ComHIP), which has been introduced in the peri-urban Lower Maya-Krobo district in the Eastern Region of Ghana. ComHIP is a community-based program for hypertension control, based on a public-private partnership, and consisting of three parts: implementation, impact evaluation and a cost-effectiveness evaluation. The private sector is engaged through licensed chemical sellers (LCSs), who are community pharmacists. The program utilizes ICTs and task shifting to enhance the capacity of the Ghana Health Service to improve management and control of hypertension. The various components of ComHIP are presented in Fig. 1.

ComHIP is also a public-private partnership between the Ghana Health services, FHI360 and the Novartis Foundation, supported by the Ghana School of Public Health and the London School of Hygiene & Tropical Medicine. While the overaching goal of ComHIP is to address the huge burden of hypertension through the deployment of a community-based model that engages the private sector public sector, and utilizes information and communication technologies (ICTs). Such a partnership plus the deployment of innovative technology increases the odds of the project’s success. The current baseline study presents hypertension prevalence, awareness, treatment, as well as knowledge and the risk factors associated with hypertension in two Ghanaian health districts.

## Methods

### Study design

To evaluate the project, a quasi-experimental study consisting of two parts (cross-sectional surveys, and a cohort study) were employed. The cross-sectional surveys constitute a before and after evaluation at baseline and endline in the intervention and control districts. The cohort study is currently being implemented in the intervention district.

### Setting

#### Intervention district

**Fig. 2:**
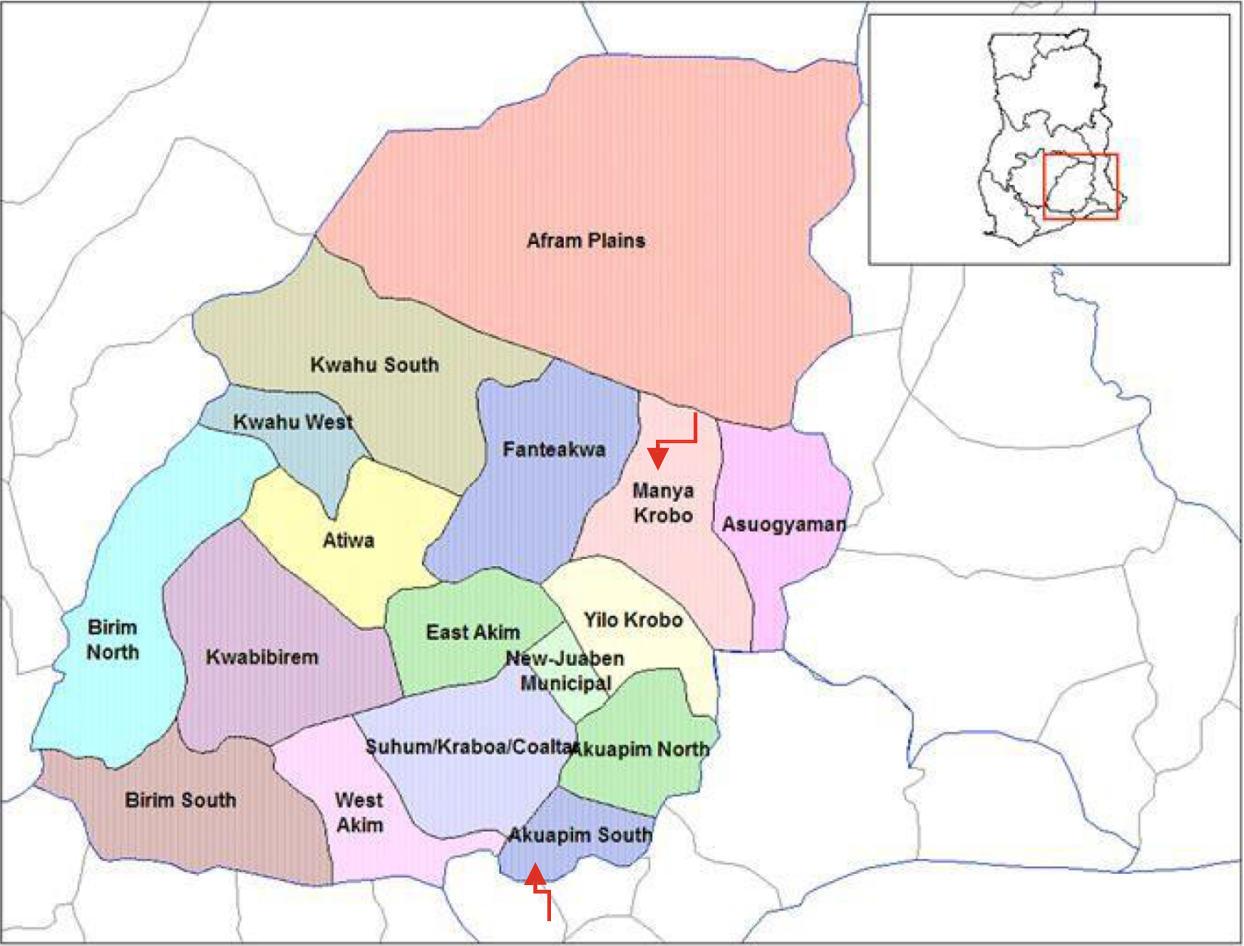
Map of the Eastern Region of Ghana highlighting the lower Manya Krobo district and Akuapim South municipality

The community selected for the intervention is Lower Manya-Krobo District in the Eastern Region of Ghana (Fig. 2). This site was selected following the unmet need of hypertension and the existing relationship of FHI360 with the district health authorities and the district authorities’ commitment to address CVD. A recent unpublished situational analysis of hypertension in that same district identified hypertension to be the fourth reason of hospital admissions in 2014 and the eighth cause of mortality. The analysis further showed that four deaths were recorded in hospital for every 100 patients admitted with hypertension [19].

The district has a population of 89,246 of whom 84% lives in urban areas. The population is served by two public hospitals, the Atua Government Hospital and Akuse Hospital, as well as a mission hospital in Agormanya, St. Martin’s. There are four public health centers, serving as primary contact points for health care, referring patients to hospitals. The district includes six sub-districts, 14 Community-based Health Planning and Services (CHPS) zones. There are approximately 75 licensed chemical sellers (LCS) in the district.

#### Comparison district

The comparison district is Akuapem South Municipality (now Nsawam-Adoagyiri Municipality and Akwapim South District), selected for its relative similarity with the intervention district regarding socio-demographic characteristics (income, employment, education); infrastructure (roads, clinics, hospitals, distance from metropolitan area); baseline health profile (disease prevalence, incidence, morbidity and mortality); development activity (private investment, NGO activity, state demonstrations projects); and level of support from the local health authority.

According to the 2010 Population and Housing Census, Akwapim South Municipality has an estimated population of 123,501, representing 4.7% of the total population of the Eastern Region. Health facilities in the municipality are the District hospital, six Health Centres, two private hospitals, five Functional Community Health Planning and Services (CHPS) compounds; 35 Demarcated CHPS compounds, two Mission Hospitals; and one Orthopedic Centre.

#### Sample size, participants sampling and summary of field procedures

For sample size calculations different assumptions about baseline prevalence, and intervention effects on different outcomes can produce different sample sizes. The main expected outcome of the ComHIP study is to reduce the proportion of people with uncontrolled hypertension. The power and sample sizes were thus calculated for reductions of prevalence of uncontrolled hypertension of about 3% in the control group and 10% in the intervention group (and for other values). Table 1 shows the power for the detection of different effect sizes on the reduction of hypertension.

**Table 1:** Sample size and power calculations for reduction in hypertension under different assumptions of baseline prevalence, intervention effect and sample size (per arm per survey)

Baseline prevalence				30%	30%	30%	35%	35%	35%
Improvement in control (A)				−3.0%	−3.0%	−3.0%	−3.5%	−3.5%	−3.5%
Improvement in intervention (B)				−9.0%	−10.5%	−12.0%	−10.5%	−12.3%	−14%
Intervention effect size (B – A):				-6%	−8%	−9%	−7%	−9%	−11%
Power if sample size = 800				51%	71%	86%	61%	81%	93%
Power if sample size = 900				56%	76%	90%	66%	85%	95%
Power if sample size = 1000				60%	80%	92%	71%	88%	97%
Power if sample size = 1100				64%	84%	94%	75%	91%	98%
Power if sample size = 1200				68%	87%	96%	78%	93%	99%
Using type I error in the analysis: alpha=				0.05	0.05	0.05	0.05	0.05	0.05
Expected baseline prevalence in both groups: A=				30%	30%	30%	35%	35%	35%
Expected improvement in control group: B=				−3.0%	−3.0%	−3.0%	−3.5%	−3.5%	−3.5%
Expected improvement in intervention group: C=				−9.0%	−10.5%	−12.0%	−10.5%	−12.3%	−14.0%
Therefore the effect of Intervention is: C-B =				-6%	−8%	−9%	−7%	−9%	−11%
Sample per group per period	1000	1.2	833	53%	72%	87%	63%	82%	94%
Sample per group per period	1100	1.2	917	57%	76%	90%	67%	86%	96%
Sample per group per period	1200	1.2	1000	60%	80%	92%	71%	88%	97%
Sample per group per period	1000	1.4	714	47%	66%	82%	56%	76%	90%
Sample per group per period	1100	1.4	786	50%	70%	85%	60%	80%	92%
Sample per group per period	1200	1.4	857	54%	74%	88%	64%	83%	94%
	(A)	(B)	(C)						

(A) = Number sampled; (B) = Desgin effect; (C) = Efective sample size

Within each of the study districts, a random sample of households was conducted using a four- stage sampling procedure. First, a purposive sampling technique was used to select the intervention and comparison districts; the second stage entailed a systematic selection of Census Enumeration Areas (EAs) from those two districts. That selection was done by the Ghana Statistical Service (GSS). The third stage entailed a simple random selection of households. Households were eligible for inclusion if one or more members were aged 30 years and above and had lived in the district during the last 12 months. All adults meeting the inclusion criteria in a selected household would be interviewed; this number ranged from 1 to 9 adults per household in both districts, with a median of 1 adult per household. There were 30 EAs each in the Intervention and Comparison districts.

Surveys were conducted using an innovative mobile technology: the Open Data Kit software on mobile devices. With this technology, data were collected at the household level. Data collected included demographics, lifestyle factors, knowledge of risk factors and physical measurements.

- The primary outcomes of the baseline survey are hypertension prevalence, awareness, proportion of patients under treatment, proportion of patients who have their hypertension under control, systolic and diastolic BP values. Secondary outcomes at baseline included knowledge of hypertension risk factors.

Definitions of outcomes:

Primary outcomes

- Prevalence of hypertension: Number of individuals in district having a SBP ≥ 140 mmHg or DBP ≥90 mmHg or receiving BP-lowering treatment. High BP was further defined as
  ○ Stage one hypertension: SBP ≥ 140 and < 160 mm/Hg, or DBP ≥90 and <100 mm/HG
  ○ Stage two hypertension: SBP ≥ 160- < 180 mm/Hg or DBP ≥100- <110 mm/HG
  ○ Stage three hypertension: SBP ≥ 180 mm/Hg or DBP ≥110 mm/Hg [2]
- Hypertension awareness: Defined by an individual’s self-reporting a diagnosis of hypertension, and found to be hypertensive, or currently taking anti-hypertensive medication
- Hypertension treatment: Percent of participants with hypertension currently under treatment.
- Hypertension control: patients with controlled hypertension: individuals self-reporting taking the medicines for hypertension prescribed by a health professional, and having a BP < 140/90 mmHg.

Secondary outcomes

- Knowledge of hypertension risk factors;
  The number of risk factors that each respondent was able to correctly identify through their own recall (without having it read to them)
  - ○ having received information on prevention of hypertension was divided into four categories. 1. not received information, 2 received information in community, including from friends/family/traditional healers/street corners) 3. received information through the media, 4. received information from health centres or healthcare providers.
- Systolic and diastolic blood pressure: baseline levels (mean and median) of BP.

Demographic data

- To ensure that the two districts were comparable we looked at basic demographics, including age, marital status, religion, ethnicity, and level of education.

Associated risk factors: we analysed risk factors that could have an association with hypertension, according to the World Heart Federation roadmap for hypertension [20].

- Physical Activity: number of times per week participants reported being physically active
- Alcohol consumption: due to issues with the survey we were only able to define participants as never consuming alcohol or drinking alcohol at least once a day
- Salt consumption: as a proxy for salt consumption, we asked whether participants added salt to food at the table and whether salt is added during food preparation. Both of these were divided into the categories: Never; Rarely; Sometimes; Often and Always
- Sleep apnoea: as a proxy for sleep apnoea we included three questions: choking while sleeping, snoring while sleeping, and snorting or gasping while sleeping
- History of hypertension
- Family history of hypertension
- Being overweight: proxy indicators for being overweight included BMI, mid-upper arm circumference (MUAC), and waist hip ratio.

### Blood pressure measurement

Blood pressure was measured a minimum of three times by survey interviewers, using as fully automated, digital devices, the OMRON digital sphygmomanometer. Interviewers were trained to use the device according to the manufacturer’s recommended protocol, using recommended methods and categories from standard guidelines the World Health Organization-International Society of Hypertension Guidelines for the Management of Hypertension [21].

### Anthropometric measurements

The anthropometric measurements of weight, height, mid-upper arm circumference, waist and hip circumference were measured according to standard procedures [22].

#### Data analysis

Categorical variables were described with proportions and continuous variables are summarised with medians, means, and standard deviations. All baseline data were recorded per comparison or intervention district, as well as overall.

#### Association of variables with outcomes

We examined what characteristics of the individuals were associated with the primary and secondary outcome variables of the survey. For each potential explanatory variable we created a separate logistic regression model against the outcomes “hypertension status” and “hypertension awareness” separately. Each model was repeated unadjusted by other variables and adjusted by age, sex and district. The models for the outcome “hypertension awareness” were restricted to people with hypertension only. *P* value less than 0.05 denotes statistical significance.

## Results

### Participant characteristics

We interviewed 1200 respondents each from the comparison and intervention districts. However, after cleaning, 1170 and 1167 surveys respectively from the comparison district and the intervention district had complete data for analysis. Demographic data are presented in Table 2. Other than ethnicity, baseline characteristics were similar between districts. Participants were overwhelmingly Christian (96.0%), had a mean age of 49 years; 66.0% being between 30 and 54 years. Only 10.0% had never been married, and 25.0% had no formal education.

**Table 2:** Knowledge of risk factors of hypertension in intervention and comparison districts

Knowledge of risk factors						
	Comparison district		Intervention district		Overall	
Number of risk factors respondent knows						
0	585	63.2%	479	58.4%	1064	60.1%
1	172	18.6%	129	15.7%	301	17.3%
2	115	12.4%	141	17.2%	256	14.7%
3	35	3.8%	51	6.2%	86	4.9%
4	12	1.3%	14	1.7%	26	4.9%
5	3	0.3%	4	0.5%	26	1.5%
6	2	0.2%	1	0.1%	7	0.4%
7	0	0.0%	0	0.0%	3	0.2%
8	1	0.1%	1	0.1%	2	0.1%
Knows alcohol as a risk factor	Comparison	Intervention		Overall		
No	779	66.6%	666	57.1%	1445	61.8%
Yes	146	12.5%	154	13.2%	300	12.8%
N/A	245	20.9%	347	29.7%	592	25.3%
Knows lack of exercise as a risk factor						
No	891	76.1%	775	66.4%	1666	71.3%
Yes	34	2.9%	45	3.9%	79	3.4%
N/A	245	20.9%	347	29.7%	592	25.3%
Knows lack of fruit as a risk factor						
No	917	78.4%	806	69.1%	1723	73.7%
Yes	8	0.7%	14	1.2%	22	0.9%
N/A	245	20.9%	347	29.7%	592	25.3%
Knows lack of vegetable as a risk factor						
No	917	78.4%	805	69.0%	1722	73.7%
Yes	8	0.7%	15	1.3%	23	1.0%
N/A	245	20.9%	347	29.7%	592	25.3%
Knows salt as a risk factor						
No	830	70.9%	719	61.6%	1549	66.3%
Yes	95	8.1%	101	8.7%	196	8.4%
N/A	245	20.9%	347	29.7%	592	25.3%
Knows obesity as a risk factor						
No	896	76.6%	794	68.0%	1690	72.3%
Yes	29	2.5%	26	2.2%	55	2.4%
N/A	245	20.9%	347	29.7%	592	25.3%
Received messages about heart health						
Received none	320	34.7%	336	41.9%	656	38.0%
In community	108	11.7%	92	11.5%	200	11.6%
Through media	319	34.6%	207	25.8%	526	30.5%
Health centre or doctor	176	19.1%	167	20.8%	343	19.9%
How important to you is lowering the salt/sodium in your diet						
Not at all important	111	9.5%	134	11.5%	245	10.5%
Somewhat important	217	18.6%	260	22.4%	477	20.5%
Very important	836	71.8%	769	66.1%	1605	69.0%

#### Prevalence of hypertension

Table 3 summarizes the prevalence, awareness, treatment and control of hypertension in the intervention and comparison districts. The prevalence of hypertension was 32.0% among women and 33.0% among men (not presented in Table 3). Overall 32.4% of the 2337 individuals were hypertensive: 18.9% of them presented with stage one hypertension, 8.2% with stage two and 4.9% with stage three. Two individuals for whom DBP was recorded to be higher than SBP were excluded.

**Table 3:** Hypertension prevalence, awareness, treatment and control in the intervention and comparison districts

	Comparison			Intervention			Total		
	N	%	95% CI	N	%	95% CI	N	%	95% CI
Examined	1170	100%		1167	100%		2337	100%	
Normal	805	68.8%	(66% – 71.4%)	785	67.3%	(64.5% – 69.9%)	1590	68.0%	(66.1% – 69.9%)
Stage 1	232	19.8%	(17.6% – 22.3%)	210	18.0%	(15.9% – 20.3%)	442	18.9%	(17.4% – 20.6%)
Stage 2	86	7.4%	(6% – 9%)	105	9.0%	(7.4% – 10.8%)	191	8.2%	(7.1% – 9.4%)
Stage 3	47	4.0%	(3% – 5.3%)	67	5.7%	(4.5% – 7.3%)	114	4.9%	(4.1% – 5.9%)
Examined	1170			1167			2337		
Hypertensive (1)	367	31.4%	(28.7% – 34.1%)	390	33.4%	(30.7% – 36.2%)	757	32.4%	(30.5% – 34.3%)
Aware (2)	164	44.7%	(39.5% – 49.9%)	186	47.7%	(42.7% – 52.8%)	350	46.2%	(42.6% – 49.9%)
Treated (2)	14	3.8%	(2.2% – 6.5%)	17	4.4%	(2.6% – 7%)	31	4.1%	(2.8% – 5.8%)
% of Aware		8.5%	(4.9% – 14.2%)		9.1%	(5.6% – 14.5%)		8.9%	(6.2% – 12.5%)
Controlled (2)	2	0.5%	(0.1% – 2.2%)	8	2.1%	(1% – 4.2%)	10	1.3%	(0.7% – 2.5%)
% Treated		14.3%	(2.5% – 43.8%)		47.1%	(23.9% – 71.5%)		32.3%	(17.3% – 51.5%)

Comparison districts: (1) % over examined individuals, (2) % over hypertensive individuals

#### Hypertension awareness

Participants aware of their hypertension are represented in Table 3. A total of 46.2% of the hypertensive individuals were aware of their condition (44.7% in the comparison district and 47.7% in the intervention district) (Table 3).

#### Hypertension treatment and control

The proportion of patients with hypertension who were under treatment is alarmingly low: only between 5 and 14% of hypertension patients in the comparison district, and between 5.6 and 14.5% in the intervention district reported to be treated for hypertension. Of those, the individuals who had their hypertension under control was 1.3% (95% CI 0.7% - 2.5%), with only 0.5% of the patients in the comparison district (0.1–2.2%) and 2.1% of the patients in the intervention district (1–4.2%) controlled for hypertension. For those patients who reported to be currently treated, hypertension control was higher at 32.3% (14.3% in the comparison site, and 47.1% in the intervention site). However, the number of patients under treatment was very low, while the 95% confidence intervals are wide and overlapping (Table 3).

### Knowledge and prevalence of biological and behavioural risk factors for hypertension

Tables 2 3, and 4 present knowledge and prevalence of risk factors for hypertension, prevalence of selected behavioral and biological risk factors of hypertension, as well as other variables. Only 5.7% of the participants from the comparison district, and 8.6% in the intervention district knew three or more of the eight known risk factors. The proportion of people who were aware of one risk factor was 19% in the comparison district, and 16% in the intervention district, while 63% in the comparison and 58% in the intervention district unfortunately knew none of the risk factors (Table 3). Participants’ knowledge of alcohol consumption, lack of exercise, and insufficient fruit and vegetables as risk factors for hypertension are presented in Table 3. Participants from both intervention and comparison districts demonstrated similarly low knowledge of the behavioural risk factors for hypertension.

**Table 4:** Prevalence of selected behavioral and biological risk factors of hypertension

Physical activity						
Times per week	Comparison		Intervention		Overall	
0	711	62.2%	679	61.1%	1390	61.6%
1	56	4.9%	46	4.1%	102	4.5%
2	60	5.2%	41	3.7%	101	4.5%
3	65	5.7%	76	6.8%	141	6.3%
4	43	3.8%	28	2.5%	71	3.2%
5	61	5.3%	61	5.5%	122	5.4%
6	36	3.2%	40	3.6%	76	3.4%
7	112	9.8%	140	12.6%	252	11.2%
Alcohol consumption						
Never Drink	1032	88.7%	1029	90.1%	2061	89.5%
Drink once a day+	131	11.3%	112	9.8%	243	10.6%
Salt						
Do you add salt to food at table						
Never	805	68.8%	581	49.8%	1386	59.3%
Rarely	54	4.6%	94	8.1%	148	6.3%
Sometimes	196	16.8%	297	25.5%	493	21.1%
Often	72	6.2%	120	10.3%	192	8.2%
Always	43	3.7%	75	6.4%	118	5.1%
Is salt added during food preparation						
Never	59	5.0%	18	1.5%	77	3.3%
Rarely	17	1.5%	11	0.9%	28	1.2%
Sometimes	47	4.0%	42	3.6%	89	3.8%
Often	247	21.1%	200	17.1%	447	19.1%
Always	800	68.4%	896	76.8%	1696	72.6%
Snoring while sleeping						
Rarely or never	947	82.4%	908	80.2%	1855	81.3%
More than once a week	202	17.6%	224	19.8%	426	18.7%
*Total 2281						
Snorting or gasping while sleeping						
Rarely or never	1027	89.1%	971	86.5%	1998	87.8%
More than once a week	126	10.9%	152	13.5%	278	12.2%
*total 2276						
Any sleep problem						
Rarely or never	928	80.0%	891	77.8%	1819	78.9%
More than once a week	232	20.0%	254	22.2%	486	21.1%
Previous history						
Any previous diagnosis of hypertension						
None	869	74.3%	71.2%	1700	72.7%	
Previous diagnosis	275	23.5%	27.4%	595	25.5%	
Do not know	24	2.1%	1.3%	39	1.7%	
No response	2	0.2%	0.1%	3	0.1%	
Family history High Blood Pressure						
No	669	57.2%	570	48.8%	1239	53.0%
Yes	364	31.1%	460	39.4%	824	35.3%
Not Sure	137	11.7%	137	11.7%	274	11.7%
BMI						
Mean	25.4	(25.1, 25.8)	25.5	(25.2, 25.9)	25.5	
Median	24.57		24.54		24.6	
Underweight	85	7.3%	85	7.3%	170	7.3%
Normal	529	45.2%	540	46.3%	1069	45.7%
Overweight	336	28.7%	321	27.5%	657	28.1%
Obese	220	18.8%	221	18.9%	441	18.9%

In Table 2, prevalence of behavioral and biological risk factors for hypertension are summarized. The majority of the participants was identified as non- alcohol users (88.7% in the intervention district and 90.1% in the control district). BMI was normal in 45.2% of the participants in the intervention district and 46.3% in the comparison district, while 7.3% of the respondents from both sites was underweight and 19.0% obese.

### Biological and behavioural risk factors for hypertension

**Fig. 3:**
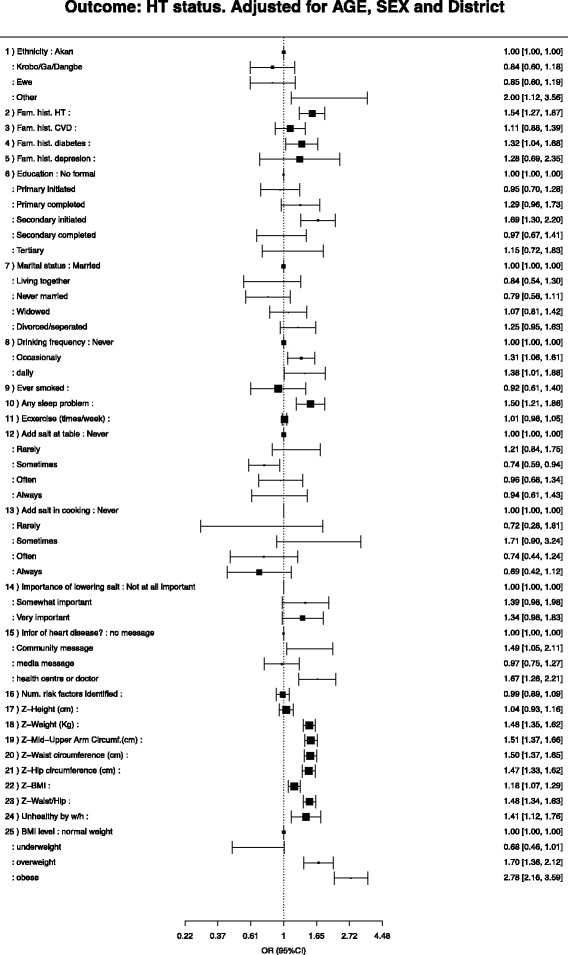
Factors associated with hypertension status, adjusted for age, sex and district

**Fig. 4:**
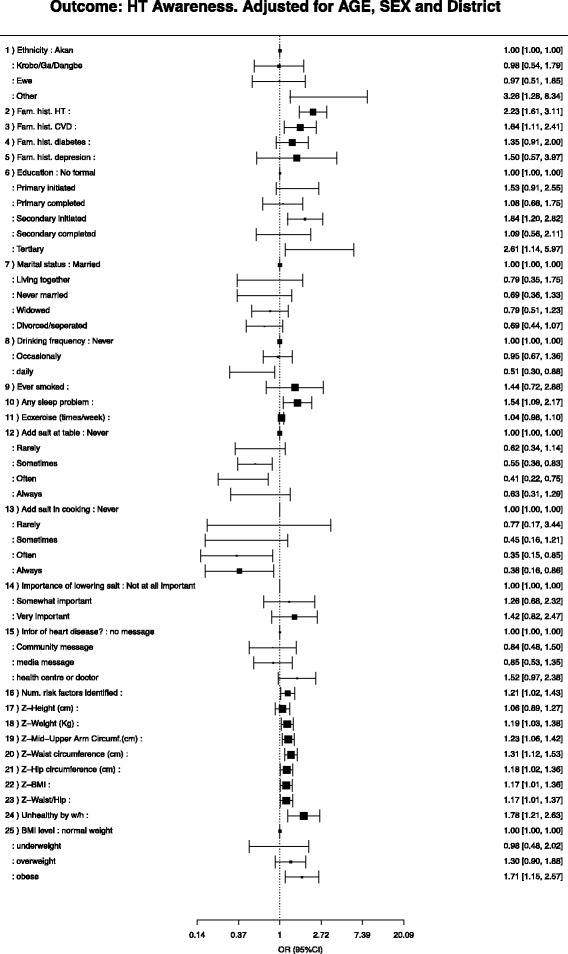
Associations between hypertension risk factors and hypertension awareness, adjusted by age, sex and district

**Fig. 5:**
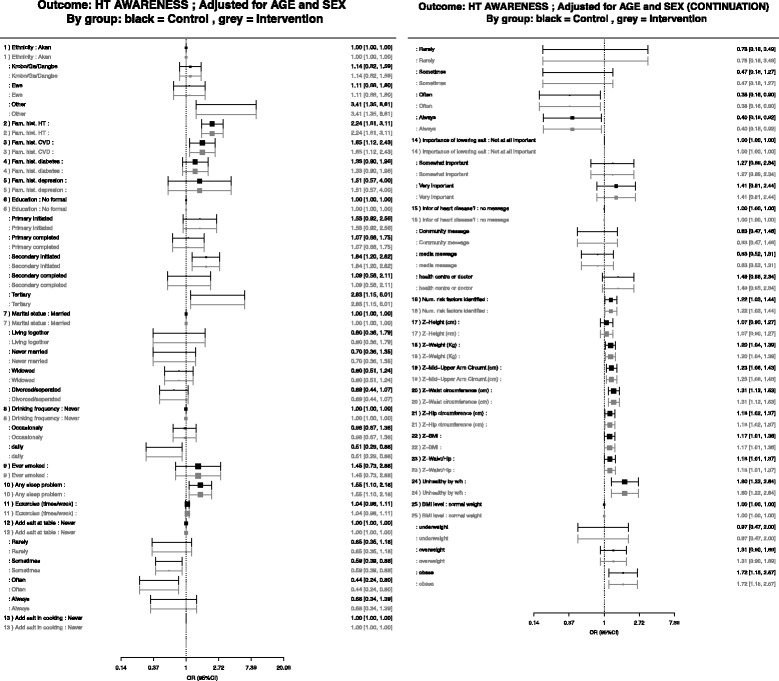
Factors associated with hypertension awareness, adjusted by age and sex divided by district

Figures 3, 4 and 5 represent the different factors associated with hypertension. Bivariate/unadjusted regression estimates are presented together with measures adjusted for age, gender and district. Age was a predictor of having hypertension; similarly, the odds of being hypertensive tended to increase with increasing BMI and waist- and hip circumference. Those with a family history of hypertension or NCD and participants who smoked also had higher odds of being hypertensive. The multiple logistic regression model examined predictors of hypertension after adjusting for a number of covariates. When adjusted for age, risk factors with the greatest association with hypertension were being overweight (aOR = 2.30; 95% CI 1.53–3.46) or obese (aOR = 3.61; 95% CI 2.37–5.51), although significant associations were also observed with increased waist/hip ratio (aOR = 1.43; 95% CI 1.30–157), MUAC (aOR = 1.47; 95%CI 1.34–1.62), high waist circumference (aOR = 1.47; 95% CI 1.34–1.61), and having some sort of sleep disturbance (aOR = 1.51; 95% CI 1.21–1.87) (Fig. 3).

Figure 4 presents factors associated with hypertension awareness, adjusted for age. In unadjusted models, older individuals were more likely to be aware of their hypertension status than younger people. When adjusted for age, sex, and district people with a family history of hypertension (aOR = 2.98; 95% CI 1.20–7.40), or CVD (aOR = 1.71; 95% CI 1.17–2.52), or with a high waist hip ratio (aOR = 1.94; 95% CI 1.44–2.63), were more likely to be aware of their hypertension status. Figure 5 compares the primary outcome of hypertension awareness, adjusted by age. There were no significant differences for the primary outcome hypertension awareness by study site.

## Discussion and conclusions

This baseline survey aimed to assess the prevalence, awareness, and risk factors of hypertension in two Ghanaian districts prior to the implementation of a community-based intervention to improve the management of hypertension. Prevalence of hypertension in the comparison district was 31.4% (95% CI 28.7.0–34.1) and 33.4% (95% CI 30.7–36.2) in the intervention district. Less than 50% of the patients with hypertension were aware of their condition and the proportion of patients under treatment was alarmingly low. Both the level of hypertension control and in-depth knowledge of the risk factors for hypertension were very low (Tables 2 and 3).

### Study site differences

Overall the findings in our two study sites were very similar. Descriptive analysis suggested that individuals in the intervention district may have had more exposure to educational information about heart health, and an overall greater awareness of risk factors. This may be because of FHI 360 s previous efforts in the area as in 2009 a facility- and community-based CVD prevention and screening project was implemented in the district hospital aiming to strengthen the capacity of facilities to manage CVD. The project also implemented facility and community-based primary and secondary prevention activities, and engaged policy makers in the prevention of CVD and NCDs in general. mHealth technology provided patients with information on healthy lifestyles and adherence to NCD treatment [18].

The largest demographic difference between the comparison and intervention sites was ethnic group, with the majority in the comparison district defining themselves as Akan, and of the majority in the intervention group as Krobo/Ga/Dangbe. Although belonging to two distinct ethnic groups, both are located within the same ecological zone and do not differ significantly in their health seeking behaviour or practices [23, 24].

### Participant characteristics

In line with the Ghana National Strategy for the Management, Prevention and Control of Chronic Non-Communicable Diseases 2012–2016, this study focused on adults aged 30 years or more. The PURE study included a comparable age group between 35 and 70 years [15] coming from both high and low-and middle income countries. Other previous studies [10–12] all involved Ghanaian men and women aged between 25 and 102 years. The 2014 Ghana Demographic and Health Survey (DHS) was a representative survey looking at hypertension [13]: although the survey is focusing on 15–49 year olds, the age band 30–49 years permits comparisons with our study. Our prevalence findings of 20.1/19.6% males/females with hypertension is similar to that found in the DHS of 23.5/20.1% males/females. The estimated hypertension prevalence in our baseline survey is comparable with that of previous local and international studies. The PURE study [15] reported an overall prevalence of 40.8% (40.7% for HICs, 49.7% for UMICs, 39.9% for LMICs, and 32.2% for LICs), while hypertension prevalence from local studies involving men and women in similar age groups were comparable with our findings (Table 3). Our prevalence estimates are substantially higher than the most recent DHS (2014) survey and lower than those found by Lloyd-Sherlock [17]. This is, however, not surprising given that the DHS survey included individuals for 15–49 and the Lloyd-Sherlock [17] only included adults over 50. Our data compare with those of a 2009 facility- and community-based CVD prevention and screening project implemented in the intervention district: about one-in-five (22%) of those screened were pre-hypertensive (systolic 120–139, diastolic 80–89) and 33% were hypertensive (systolic ≥140, diastolic ≥90) [18]). Our estimated prevalence of hypertension is consistent with reported prevalence in other parts of Africa [8, 25], such as in the neighboring cities of Abidjan in 2005 and Cotonou in 2007, the prevalence was 21.7% and 27.3% respectively [26]. Higher prevalence was however reported in semi-urban Nigeria (37%) [25], Burkina Faso (40%) [27] and Niger (42%) [26].

We estimated that only between 42.6 and 50% of the people with hypertension were aware of their condition, while PURE reported an overall awareness of 46.5% (49.0% for HICs, 52.5% for UMICs, 43.6% for LMICs, and 40.8% for LICs). The level of awareness in our study was higher than in most previous studies in Ghana (Table 5), but disparity in age, as well as setting peculiarity may explain the differences.

**Table 5:** Demographic characteristics of participants in intervention and comparison districts

	Comparison		Intervention		Overall	
Demographics	1170		1167		2337	
Numbers in survey						
Age		*std dev*		*std dev*		*std dev*
Mean	49.6	14.9	49.4	15.5	49.5	15.2
Median	47		46		46	
Number of adults per age category						
30–44	508	43.4%	518	44.4%	1026	43.9%
45–54	264	22.6%	239	20.5%	503	21.5%
55–64	186	15.9%	193	16.5%	379	16.2%
65+	212	18.1%	217	18.6%	429	18.4%
# Adults per HH						
Range	1, 9		1, 9		1,9	
Mean	1.6		1.3		1.4	
Median	1		1		1	
Sex						
% male	35.5%		36.5%		36.0%	
Marital status						
Never married	117	10.0%	152	13.0%	269	11.5%
Living together	59	5.0%	72	6.2%	131	5.6%
Married	647	55.3%	641	54.9%	1288	55.1%
Widowed	166	14.2%	179	15.3%	345	14.8%
Divorced/separated	181	15.5%	123	10.5%	304	13.0%
Religion						
Christian	1120	95.7%	1119	95.6%	2239	95.8%
Muslim	12	1.0%	21	1.8%	33	1.4%
Traditional/Spiritualist	13	1.1%	15	1.3%	28	1.2%
No religion	18	1.5%	9	0.8%	27	1.2%
Other	7	0.6%	3	0.3%	10	40.0%
Ethnicity						
Akan	945	80.8%	47	4.0%	992	42.5%
Krobo/Ga/Dangbe	78	6.7%	882	75.6%	960	41.1%
Ewe	113	9.7%	218	18.7%	331	14.2%
Grussi	13	1.1%	2	0.2%	15	0.6%
Mole-Dagbani	3	0.3%	3	0.3%	6	0.3%
Hausa	5	0.4%	8	0.7%	13	0.6%
Other	13	1.1%	7	0.6%	20	0.9%
Level of education						
No formal education	285	24.4%	314	26.9%	599	25.6%
Less than Primary	202	17.3%	182	15.6%	384	16.4%
Completed primary	210	18.0%	196	16.8%	406	17.4%
Some secondary	315	26.9%	283	24.3%	598	25.6%
Completed secondary	105	9.0%	117	10.0%	222	9.5%
Tertiary Completed post- graduate	50	4.3%	73	6.3%	123	5.3%
Not sure	2	0.2%	0	0.0%	2	0.1%

Compared to PURE, the overall self-reported treatment and control rates in our study were only 31 of 757 hypertensive individuals (14 from the comparison district, and 17 from the intervention district). Among the treated hypertension patients, the control rate was 9% overall, with 14.3% in the control arm and 47.1% in the intervention. In the Ghana DHS only 11.5% of hypertensives under treatment were reported to have their hypertension under control [13].

### Associations with risk factors

Supportive of previous studies [10–12, 28], our study showed that age, BMI, and biological factors were positively associated with both hypertension status and awareness of the condition. Older individuals, females, people with a family history of CVD and with unhealthy hip/waist ratios were more likely to be aware of their hypertension status. Age, increasing BMI and waist hip circumference were predictors of having hypertension.

This was a community-based study with a sample size of over 2000 individuals, one of the largest studies of its kind in the region. This study is part of a larger, mixed-methods evaluation of community based hypertension control project, and will be followed up with a second similar sized cross sectional survey. However, as with any survey there are some potential limitations of the data collected. Due to safety issues data collectors were only able to visit households during daylight hours, and as a result some adults were at work when they visited the house. To minimize this, collectors would come back to houses on multiple occasions. This issue seems however to be similar in both the comparison and intervention districts.

### Study implications and future research

The results of this baseline survey have implications for the intervention being implemented and for future research. The high burden of hypertension among the studied population, coupled to the relatively high level of awareness yet very low levels of hypertension treatment and control requires in-depth investigation of the bottlenecks to treatment and control. Secondly, the low treatment and control rates despite current and previous general educational programs in the intervention district, are suggestive that such programs are not really impactful health service delivery strategies. The RODAM study reported hypertension prevalence among rural Ghanaian women/men to be 32.1% 25.5% respectively, and higher among urbanized populations (37.9% 38.2% respectively) [29]. The progressive rise of hypertension as Ghanaians become more urbanized in Berlin, London and Amsterdam, even in the presence of more sophisticated health care, show that Ghanaians continue to be at risk of uncontrolled hypertension. It may therefore be argued that attitude/behaviour is as important as access, if not more so.

These are important findings for the ongoing intervention. The lack of in-depth knowledge of hypertension risk factors and the high burden of biological risk factors of hypertension, particularly obesity, will be notable challenges for the intervention to have its desired impact.

The project’s next steps include an in-depth mapping of individuals with hypertension by census enumeration areas. The key findings of the survey have been shared with the program Technical and Steering Committees, to optimize targeting of interventions. The implementation of the program is currently being carried out. The endline survey will be conducted in 2017, to evaluate the change in hypertension prevalence, awareness, treatment and control between the two districts. Using individual patient data, we will apply logistic regressions for each of these outcomes including the district and survey period baseline / endline variables and look at their interaction; we will also adjust for age. Given that the observed awareness at baseline in either group was about 46%, a sample size of 1150 individuals in each group in the end line survey will provide about a 85% power to detect a difference of 10% between groups. It will also provide a more than 90% power to detect a difference of 7% in treatment coverage in people aware of hypertension (versus a baseline of about 9%), and a 87% power to detect a difference of 10% in the proportion of patients under treatment who have their BP under control (versus a baseline of about 32%).

## Additional files

Additional file 1: Unadjusted associations between various factors and hypertension. (DOCX 332 kb)

Additional file 2: Unadjusted association between hypertension awareness and risk factors. (DOCX 332 kb)

## Abbreviations

BMI: Body mass index
CHO: Community health officer
CHPS: Community-based health planning and services
ComHIP: Community-based hypertension improvement programme
CVD: Cardiovascular disease
DBP: Diastolic blood pressure
DSS: Decision support system
EAs: Census enumeration areas
FHI360: Family Health International 360
GHS: Ghana Health Service
GSS: Ghana Statistical Service
HPT: Hypertension/high blood pressure
ICC: Intra class correlation
ICT: Information and communication technologies
IRBs: Institutional Review Boards
LCS: Licensed chemical seller
LMIC: Low- and middle-income countries
LMK: Lower Manya Krobo
LSHTM: London School of Hygiene and Tropical Medicine
MIC: Middle income country
MOH: Ministry of health
MUAC: Mid upper arm circumference
NCDCP: NCD Control Programme
NCDs: Non-communicable diseases
ODK: Open Data Kit
PURE: The prospective urban rural epidemiology study
SBP: Systolic blood pressure
UIC: Upper income country
WHO: World Health Organization

## References

[1] Forouzanfar M, Dajani HR, Groza VZ, Bolic M, Rajan S, Batkin I (2015). Oscillometric blood pressure estimation: past, present, and future. IEEE Rev Biomed Eng.

[2] Organization WH (1996). Hypertension control: report of a WHO expert committee.

[3] Sundstrom J, Arima H, Woodward M, Jackson R, Karmali K, Lloyd-Jones D, Baigent C, Emberson J, Rahimi K, Blood Pressure Lowering Treatment Trialists C (2014). Blood pressure-lowering treatment based on cardiovascular risk: a meta-analysis of individual patient data. Lancet.

[4] Collins R, Peto R, MacMahon S, Hebert P, Fiebach NH, Eberlein KA, Godwin J, Qizilbash N, Taylor JO, Hennekens CH (1990). Blood pressure, stroke, and coronary heart disease. Part 2, short-term reductions in blood pressure: overview of randomised drug trials in their epidemiological context. Lancet.

[5] Law MR, Morris JK, Wald NJ (2009). Use of blood pressure lowering drugs in the prevention of cardiovascular disease: meta-analysis of 147 randomised trials in the context of expectations from prospective epidemiological studies. BMJ.

[6] Khor GL (2001). Cardiovascular epidemiology in the Asia-Pacific region. Asia Pac J Clin Nutr.

[7] Vorster HH (2002). The emergence of cardiovascular disease during urbanisation of Africans. Public Health Nutr.

[8] Addo J, Smeeth L, Leon DA (2007). Hypertension in sub-saharan Africa: a systematic review. Hypertension.

[9] Ministry of Health. The Ghana Health Sector 2006 Programme Of Work, 2005. Accra: Ministry of Health; 2005.

[10] Addo J, Agyemang C, Smeeth L, de Graft Aikins A, Edusei AK, Ogedegbe O (2012). A review of population-based studies on hypertension in Ghana. Ghana Med J.

[11] Amoah AG (2003). Hypertension in Ghana: a cross-sectional community prevalence study in greater Accra. Ethn Dis.

[12] Cappuccio FP, Micah FB, Emmett L, Kerry SM, Antwi S, Martin-Peprah R, Phillips RO, Plange-Rhule J, Eastwood JB (2004). Prevalence, detection, management, and control of hypertension in Ashanti, West Africa. Hypertension.

[13] GSS, GHS, ICFI (2015). Ghana demographic and health survey 2014.

[14] Bosu WK (2010). Epidemic of hypertension in Ghana: a systematic review. BMC Public Health.

[15] Chow CK, Teo KK, Rangarajan S, Islam S, Gupta R, Avezum A, Bahonar A, Chifamba J, Dagenais G, Diaz R (2013). Prevalence, awareness, treatment, and control of hypertension in rural and urban communities in high-, middle-, and low-income countries. JAMA.

[16] Adeloye D, Basquill C (2014). Estimating the prevalence and awareness rates of hypertension in Africa: a systematic analysis. PLoS One.

[17] Lloyd-Sherlock P, Beard J, Minicuci N, Ebrahim S, Chatterji S (2014). Hypertension among older adults in low- and middle-income countries: prevalence, awareness and control. Int J Epidemiol.

[18] FHI360 (2012). Descriptive analysis of NCD risk factors in three communities in Ghana: a pilot study.

[19] Lower Manya Krobo Municipal Health Directorate. Lower Manya Krobo municipal situational analysis of Hypertension. Atua, Ghana. 2014.

[20] Adler AJ, Prabhakaran D, Bovet P, Kazi DS, Mancia G, Mungal-Singh V, Poulter N (2015). Reducing cardiovascular mortality through prevention and management of raised blood pressure: a world heart federation roadmap. Glob Heart.

[21] WHO. 1999 World Health Organization-International Society of Hypertension Guidelines for the Management of Hypertension. Guidelines Subcommittee. Am J Hypertens. 1999;17(2):151–83. 10067786

[22] Jelliffe DB (1966). The assessment of the nutritional status of the community (with special reference to field surveys in developing regions of the world). Monogr Ser World Health Organ.

[23] Yarney L, Mba C, Asampong E (2015). Qualitative study on the socio-cultural determinants of care of children orphaned by AIDS in the Ashanti and Eastern regions of Ghana. BMC Public Health.

[24] Geest S, Krause K (2014). Introduction: studying health and health care in Ghana. Ghana Stud.

[25] Adedoyin RA, Mbada CE, Balogun MO, Martins T, Adebayo RA, Akintomide A, Akinwusi PO (2008). Prevalence and pattern of hypertension in a semiurban community in Nigeria. Eur J Cardiovasc Prev Rehabil.

[26] World Health Organization (2008). STEPS fact sheet. Brazzaville: WHO AFRO 2008.

[27] Niakara A, Fournet F, Gary J, Harang M, Nebie LV, Salem G (2007). Hypertension, urbanization, social and spatial disparities: a cross-sectional population-based survey in a West African urban environment (Ouagadougou, Burkina Faso). Trans R Soc Trop Med Hyg.

[28] Bosu WK (2012). A comprehensive review of the policy and programmatic response to chronic non-communicable disease in Ghana. Ghana Med J.

[29] Agyemang C, Beune E, Meeks K, Owusu-Dabo E, Agyei-Baffour P, Aikins A, Dodoo F, Smeeth L, Addo J, Mockenhaupt FP (2014). Rationale and cross-sectional study design of the research on obesity and type 2 diabetes among African migrants: the RODAM study. BMJ Open.

