# Supplementary material for: Evaluation of a community-based hypertension improvement program (ComHIP) in Ghana: data from a baseline survey
Source: BMC Public Health. 2017 Apr 28;17:368. doi: 10.1186/s12889-017-4260-5 (PMC5410035; doi:10.1186/s12889-017-4260-5)
Supplement: Supplementary file 2 — Unadjusted association between hypertension awareness and risk factors. (DOCX 332 kb) [file 12889_2017_4260_MOESM2_ESM.docx]

**Additional file 2. Unadjusted association between hypertension awareness and risk factors**
